# Comprehensive Genome Analysis of Two Bioactive *Brevibacterium* Strains Isolated from Marine Sponges from the Red Sea

**DOI:** 10.3390/biology14091271

**Published:** 2025-09-15

**Authors:** Yehia S. Mohamed, Samar M. Solyman, Abdelrahman M. Sedeek, Hasnaa L. Kamel, Manar El Samak

**Affiliations:** 1 Department of Pathological Sciences, College of Medicine, Ajman University, Ajman P.O. Box 346, United Arab Emirates; 2 Department of Microbiology and Immunology, Faculty of Pharmacy (Boys), Al-Azhar University, Cairo 11884, Egypt; 3 Department of Microbiology and Immunology, Faculty of Pharmacy, Sinai University—Kantara Branche, Ismailia 41522, Egypt; hasnaa.kamel@su.edu.eg; 4 Department of Microbiology and Immunology, Faculty of Pharmacy, Suez Canal University, Ismailia 41522, Egypt; manarelsamak@pharm.suez.edu.eg; 5 Department of Microbiology and Immunology, Faculty of Pharmacy, Galala University, New Galala City 43511, Egypt; abdelrahman.sedek@gu.edu.eg

**Keywords:** *Brevibacterium luteolum*, *Brevibacterium casei*, microbial genomics, antimicrobial activity, sponge-associated bacteria, whole-genome sequencing, Actinomycetota

## Abstract

Marine-derived Actinomycetota have emerged as promising sources of bioactive natural products, particularly filamentous actinomycetes (e.g., *Streptomyces*). However, members from non-filamentous genera have showed potential biotechnological importance. In this study, we performed a comprehensive genomic characterization of two bioactive *Brevibacterium* strains, *Brevibacterium luteolum (B. luteolum)* 26C and *Brevibacterium casei (B. casei)* 13A, isolated from two Red Sea sponges. Whole-genome sequencing and taxonomic analysis confirmed species-level identification, marking the first documented report of these species within the Red Sea ecosystem. The two strains displayed antimicrobial activity against *Staphylococcus aureus*, *Escherichia coli*, and *Candida albicans*. Additionally, functional annotation revealed multiple genomic islands (GIs) enriched with genes conferring heavy metal resistance, DNA repair enzymes, nutrient acquisition, and mobile genetic elements, highlighting potential evolutionary adaptations to the harsh physicochemical conditions of the Red Sea. Genome mining identified biosynthetic gene clusters, including those encoding ε-poly-L-lysine, tropodithietic acid, ectoine, and carotenoids. The comparative analysis of orthologous gene clusters from both strains and their counterparts from terrestrial ecosystems highlighted potential marine adaptive genetic mechanisms. This study highlights the biosynthetic potential of *B. luteolum* 26C and *B. casei* 13A and their ecological role as active competitors and potential defensive associates within the sponge microbiome.

## Simple Summary

The Red Sea is a challenging marine environment with very harsh environmental conditions. However, its ecosystems support a rich diversity of organisms, including marine sponges that maintain close associations with diverse microbial communities. In this study, we investigated two *Brevibacterium* strains isolated from Red Sea sponges to explore their genetic adaptations to these challenging conditions and to assess their capacity for producing bioactive compounds. The metabolites from the two strains exhibited moderate antimicrobial activity. Whole-genome sequencing revealed genes associated with tolerance to salinity and nutrient limitation, as well as genetic pathways for resistance to toxic compounds. Furthermore, some biosynthetic gene clusters were identified, indicating a capacity to produce structurally diverse secondary metabolites with potential pharmaceutical and industrial applications. These findings provide new insights into the adaptive mechanisms of sponge-associated bacteria in extreme marine habitats and highlight their potential as a source of novel bioactive molecules. The study advances our understanding of microbial survival strategies in harsh marine environments and underscores the importance of such microorganisms for both ecological functions and biotechnological innovation.

## 1. Introduction

The Red Sea is a unique marine ecosystem characterized by high salinity, elevated temperatures, and oligotrophic conditions, creating a challenging yet biologically rich environment [1,2]. Its coral reefs and sponge communities harbor diverse microbial symbionts that have adapted to these challenging physicochemical stressors, making them valuable models for studying marine microbial ecology and evolution [3]. Marine sponges, in particular, are recognized as prolific sources of bioactive natural products, largely due to their complex symbiotic microbiomes [4,5]. These microbial consortia contribute to host defense, chemical communication, and environmental adaptation through the production of bioactive metabolites with antibacterial, antifungal, antiviral, and anticancer properties [6,7]. Focusing on the Red Sea sponge-associated microbiome, our lab has previously reported several microbial strains with interesting bioactivities and potential genetic adaptation mechanisms [8,9,10].

Marine Actinomycetota associated with sponges, particularly filamentous actinomycetes (e.g., *Streptomyces*), have attracted attention for their biosynthetic versatility and capacity to yield novel natural products [11,12]. However, other non-filamentous genera such as *Brevibacterium*, although less explored compared to *Streptomyces*, are increasingly reported from various marine habitats, including sediments, seawater, and sponge microbiomes. Representative species include *B. marinum* (from seawater) [13]; *B. oceani* (from deep-sea sediments, Chagos Trench in the Indian Ocean) [14]; *B. profundi* (from deep-sea sediments, Western Pacific Ocean); and *B. spongiae* (from marine sponges) [15]. *These different species* have emerged as a promising reservoir of unique bioactive compounds, including pigments and biosurfactants, with potential applications in biotechnology and bioremediation [13,14,15]. These capabilities, combined with frequent reports of occurrence in diverse marine niches, underscore the ecological adaptability and applied potential of this genus.

Whole-genome sequencing (WGS) has significantly advanced our understanding of the diverse *Brevibacterium* genus, providing valuable insights into their genetics, metabolism, and ecological roles. Excluding the genomes analyzed in this study, as of August 2025, the NCBI genome database contained 38 genomes of *B. casei* and 19 genomes of *B. luteolum*. The predominant isolation sources of these species were cheese, dairy products, fermented foods, or human/animal-associated sources. In contrast, isolates derived from marine-associated environments represent only a minor proportion, indicating their relative scarcity within current genomic datasets. Beyond basic genome statistics, WGS projects identify genes involved in essential functions like carbon utilization, nitrogen and phosphate metabolism, metal transport, and resistance to antibiotics and toxic compounds [16]. Furthermore, WGS studies have illuminated the potential of *Brevibacterium* species to produce a variety of secondary metabolites, including biosurfactants and other bioactive compounds, which hold promise for various biotechnological applications [17]. Thus, genomic analyses enhance our understanding of microbial functional capacity, ecological adaptation, and the potential for natural product discovery. This study aims to investigate the genomic features of two Red Sea sponge-associated *Brevibacterium* isolates to elucidate their biosynthetic potential, ecological adaptations, and capacity to produce biotechnologically beneficial compounds.

## 2. Materials and Methods

### 2.1. Strains Isolation and Antimicrobial Activity Screening

The strains 26C and 13A were previously isolated in our laboratory from two Red Sea sponges [9]. For metabolic extract preparation, the glycerol stocks (−80 °C) of both isolates were plated on Reasoner’s 2A agar (R2A) (Difco^TM^, Detroit, MI, USA) supplemented with 2% NaCl. The plates were incubated at 30 °C for 1–2 days. The isolates were inoculated in flasks containing 100 mL R2A broth medium made with deionized water type 1. Following 7 days of incubation at 25 °C on an incubator shaker at 220 rpm, the fermented broths were extracted twice with double volume ethyl acetate (200 mL × 2). The solvent extracts were concentrated to dryness under reduced pressure using a rotary evaporator at 40 °C and 170 mbar.

For antimicrobial activity screening, the extracted residues were dissolved in 15% dimethylsulfoxide (DMSO) at a concentration of 1 mg/mL. The standardized well-diffusion method was used to investigate the antimicrobial activity using 100 μL of each metabolic extract against Gram-negative strains (*Escherichia coli* ATCC 10536 and Pseudomonas aeruginosa ATCC25619), a Gram-positive strain (*Staphylococcus aureus* ATCC 9144), and a yeast (*Candida albicans* ATCC 90028). Additionally, 15% DMSO was used as a negative control. Ceftazidime and imipenem were used as positive controls for the Gram-negative stains, ampicillin was used as a positive control for *S. aureus* growth, and nystatin was used as a positive control for *C. albicans*.

### 2.2. DNA Extraction and Whole-Genome Sequencing

#### 2.2.1. Reads Preprocessing and Assembly

Trimmomatic version 0.39 was used for quality-based filtration of the raw reads [18]. An Illumina adaptor clipping option, sliding window trimming of a minimum of 4 bases, and an average required quality of 20 were adjusted as parameters for the filtration process. After that, the filtered reads were assembled using the Unicycler assembler [19] on the BV-BRC server (https://www.bv-brc.org) accessed on 24 May 2025.

#### 2.2.2. Strain Typing and Phylogeny

The strain typing was carried out using the GTDB-Tk version 2.1.0 toolkit against release 220 (28 October 2024) of the Genome Taxonomy Database (GTDB) [20]. A phylogenomic-based genome analysis between the genomes of isolates 26C, 13A, and top related type strains was conducted on the Type Strain Genome Server (TYGS) (Leibniz Institute DSMZ, Braunschweig, Germany) [21]. To ensure the taxonomic affiliations of both isolates, the average nucleotide identity (ANI) and DNA–DNA hybridization (DDH) were calculated using the JSpecies server (Leibniz Institute DSMZ, Braunschweig, Germany) [22] and Genome-to-Genome Distance Calculator (Leibniz Institute DSMZ, Braunschweig, Germany) [23]. The average amino acid identity (AAI) was calculated using the AAI calculator tool (Kostas Lab, Georgia Institute of Technology, Atlanta, GA, USA) [24].

#### 2.2.3. Reference-Guided Scaffolding and Genome Annotation

RagTag version 2.1.0 was used for reference-guided scaffolding using the default parameters [25]. The Rapid Annotations using Subsystems Technology (RAST) [26] and Prokka [27] were used to annotate the genomes of strains 26C and 13A. The mobile OG-db was used to annotate the bacterial mobile genetic elements (MGEs) [28]. IslandViewer 4 (Simon Fraser University, Burnaby, BC, Canada) was used to analyze the genomic islands (GIs) within the genomes of strains 26C and 13A [29].

#### 2.2.4. Metabolic Pathway Reconstruction and Investigation of Biosynthetic Gene Clusters (BGCs)

To investigate the main metabolic processes in the isolated strains, KEGG pathway annotation and reconstruction were performed using GhostKOALA version 3.1, which automatically assigned KEGG Orthology (KO) identifiers and mapped the predicted protein products from the coding sequences (CDSs) to metabolic pathways [30]. The BGCs responsible for secondary metabolite production and their similarities to known clusters were identified using antiSMASH bacterial version 8.0.1 [31].

#### 2.2.5. Comparative Orthologous Cluster Analysis

To gain an overview of potential marine adaptive genetic elements in *B. luteolum* 26C and *B. casei* 13A, a comparative orthologous cluster analysis was performed against related genomes from non-marine niches (Table 1). The analysis was conducted using the OrthoVenn3 web server (https://orthovenn3.bioinfotoolkits.net/home accessed on 30 August 2025) [32].

## 3. Results

### 3.1. The Isolated Strains’ Phenotypic Characteristics

**Figure 1:**
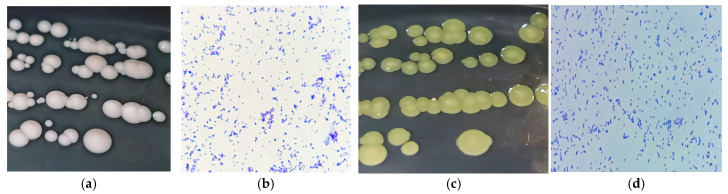
(**a**,**c**) Colony morphologies of *B. casei* 13A and *B. luteolum* 26C, respectively, on incubation on marine agar at 30 °C for 1–2 days; (**b**,**d**) microscopical examination of *B. casei* 13A and *B. luteolum* 26C, respectively.

The colony morphologies of *B. casei* 13A and *B. luteolum* 26C are shown in Figure 1a,c and described in Table 2.

The microscopical examination of *B. casei* 13A shows small Gram-positive irregular bacilli (Figure 1b), while *B. luteolum* 26C showed small Gram-positive coccobacilli (Figure 1d).

### 3.2. Antimicrobial Activities of B. luteolum 26C and B. casei 13A

The metabolic extracts of the strains 26C and 13A showed broad-spectrum antimicrobial activity against *S. aureus*, *E. coli*, and *C. albicans* (Table 3).

### 3.3. Genome Characteristics and Strain Typing

For isolate 26C, genome sequencing yielded 29 contigs with an N_50_ value of 206,763 bp, a GC content of 67.05%, and a total genome length of 2,920,920 bp. Strain typing analysis based on the relative evolutionary divergence (RED) and average nucleotide identity (ANI) criteria was conducted using GTDB-Tk against the GTDB database, which identified the isolate 26C as *B. luteolum*, a high GC-content, Gram-positive bacterium within the phylum Actinomycetota, order Micrococcales, and family Brevibacteriaceae.

**Figure 2:**
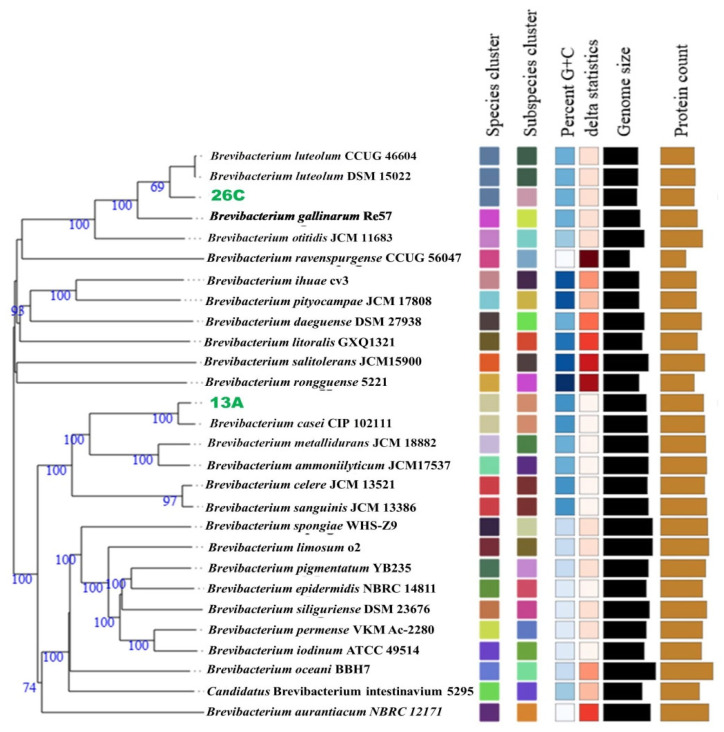
A phylogenomic tree constructed by the Type Strain Genome Server (TYGS) based on the genomes of *Brevibacterium casei* 13A, *Brevibacterium luteolum* 26C, and their top related type strains. Confidence values are displayed near the nodes.

On the other hand, the isolate 13A genome assembly resulted in 61 contigs with an N_50_ of 111,271 bp, a GC content of 68.27%, and a total size of 3,689,383 bp. GTBD-Tk classified this isolate as *B. casei*. A phylogenomic tree depicting the evolutionary relationships of both strains and their closely related type strains, made by the TYGS server, is presented in Figure 2.

To confirm the taxonomic positions of both isolates, dDDH, AAI, and ANI comparisons were performed with their closest type strains. For isolate 26C, the type strain *B. luteolum* CCUG 46604 (NCBI accession: GCF_013004595.1) exhibited the closest relationship, with dDDH and ΔCG values of 71.1% and 0.02%, respectively. The ANIm and AAI values between the genomes were 96.77% and 96.31%, respectively.

In the case of isolate 13A, the type strain *B. casei* CIP 102111 (NCBI accession: GCF_900169275.1) showed the closest relationship, with dDDH and ΔCG values of 82.4% and 0.23%, respectively. The ANIm and AAI values between these genomes were 98.13% and 97.79%, respectively. These results confirm the taxonomic affiliations of both isolates.

### 3.4. B. luteolum 26C and B. casei 13A Genome Annotation and Genome Mapping

**Figure 3:**
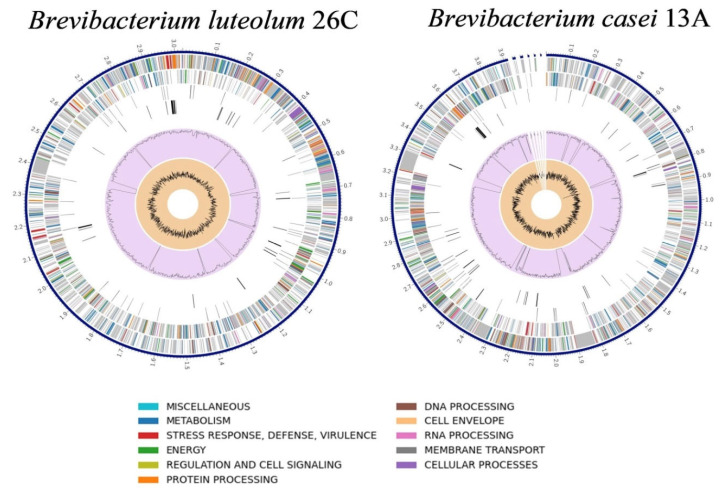
Circular diagrams representing the genome maps of *Brevibacterium luteolum* 26C and *Brevibacterium casei* 13A.

The Similar Genome Finder tool (www.bv-brc.org/app/GenomeDistance accessed on 24 May 2025) identified *B. luteolum* strain NEB1784 (GenBank: CP035810.1) as the closest complete genome to 26C, with a distance of 0.029898, making it suitable for reference-guided scaffolding. Using RagTag, the 26C genome was scaffolded into a single chromosome of 3,025,421 bp, covering 96.1% of the reference genome. On the other hand, the genome of *B. casei* FDAARGOS_1100 (GenBank: CP068173) was the closest complete genome to the genome of *B. casei* 13A, with a distance of 0.012729, so it was selected as the reference for reference-guided scaffolding. The scaffolding of *B. casei* 13A resulted in one main scaffold of 3,917,481 bp (93% coverage) and five smaller contigs ranging from 5495 to 13,337 bp. The RAST and Prokka pipelines were used to annotate the genomes of *B. luteolum* 26C and *B. casei* 13A. For both, while RAST returned more annotations with functional assignments, Prokka was able to call a greater number of annotations with EC assignments (Table 4). The genome maps of *B. luteolum* 26C and *B. casei* 13A are illustrated in Figure 3.

**Figure 4:**
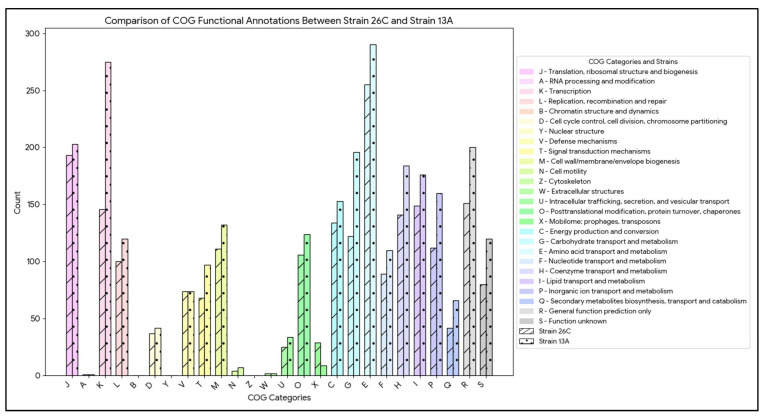
Bar chart comparing the number of genome-derived protein counts per COG functional category in *Brevibacterium luteolum* 26C and *Brevibacterium casei* 13A.

A total of 81.49% and 81.05% of genome-derived proteins were assigned to a COG functional category for *B. luteolum* 26C and *B. casei* 13A, respectively. The comparative analysis of COG functional annotations between *B. luteolum* 26C and *B. casei* 13A reveals distinct differences in their metabolic and functional capacities. Notably, *B. casei* 13A generally exhibits higher counts in all COG functional categories except the category X (Mobilome: prophages, transposons), with 29 annotations for strain 26C compared to 9 for strain 13A (Figure 4).

**Figure 5:**
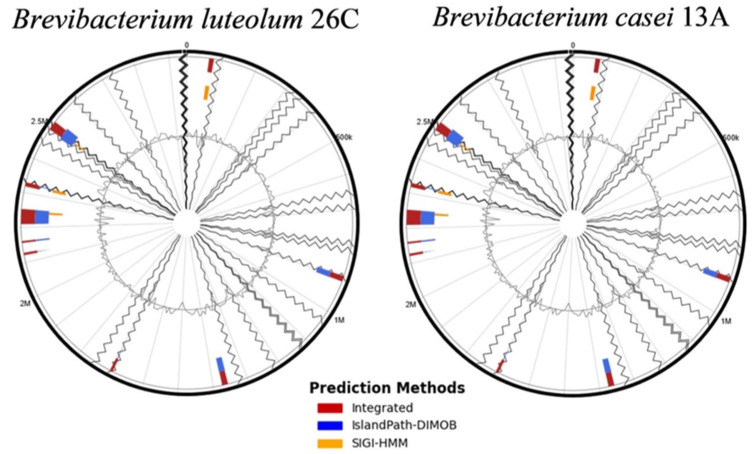
The genomic islands (GIs) identified within the genomes of *Brevibacterium luteolum* 26C and *Brevibacterium casei* 13A using the IslandViewer 4 server.

IslandViewer 4 revealed the presence of multiple genomic islands (GIs) within the genomes of both *B. luteolum* 26C (12 GIs) and *B. casei* 13A (14 GIs) (Figure 5). A consistent theme across both strains is the prevalence of genes associated with metal detoxification and resistance, including specific transporters for nickel, cadmium, cobalt, zinc, copper, arsenic, and chromate. Furthermore, both strains exhibit robust DNA repair and maintenance systems, with various glycosylases, helicases, and recombinases (Table 5 and Table 6).

Moreover, numerous mobile genetic elements (MGEs), including a variety of transposases and insertion sequences, were identified in both genomes. *B. luteolum* 26C harbors a greater number of MGE-associated genes compared to *B. casei* 13A, with a total of 67 genes versus 49, respectively (Table 7).

### 3.5. Metabolic Pathways and Biosynthetic Gene Clusters (BGCs)

**Figure 6:**
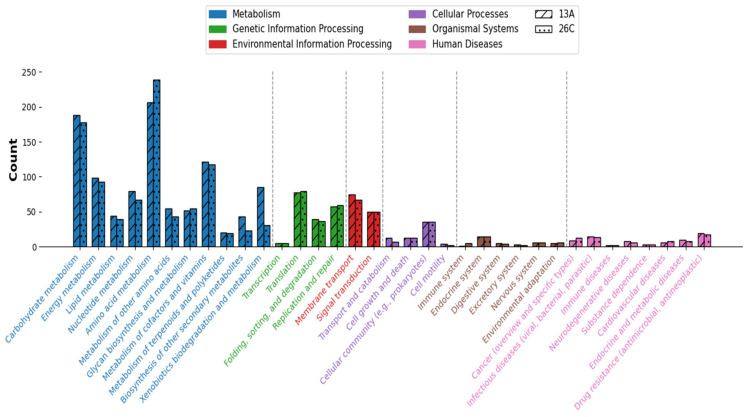
Comparison between the numbers of genes annotated under each KEGG subcategory in *Brevibacterium luteolum* 26C and *Brevibacterium casei* 13A. Subcategories under the same top-level category are colored the same.

From 3449 and 2672 CDs, there were 1552 (~45%) and 1353 (~51%) protein products annotated on the KEGG database for *B. casei* 13A and *B. luteolum* 26C, respectively. The annotated proteins were incorporated into 232 KEGG pathways for *B. casei* 13A and 221 for *B. luteolum* 26C (Figure 6).

There were a total of 44 and 51 complete KEGG modules identified within the genomes of *B. luteolum* 26C and *B. casei* 13A, respectively. While the majority of these modules were shared between both strains, there were some unique modules. For instance, the biosynthesis of ectoine (M00033) module was identified within the genome of *B. casei* 13A, while not found within the genome of *B. luteolum* 26C.

**Figure 7:**
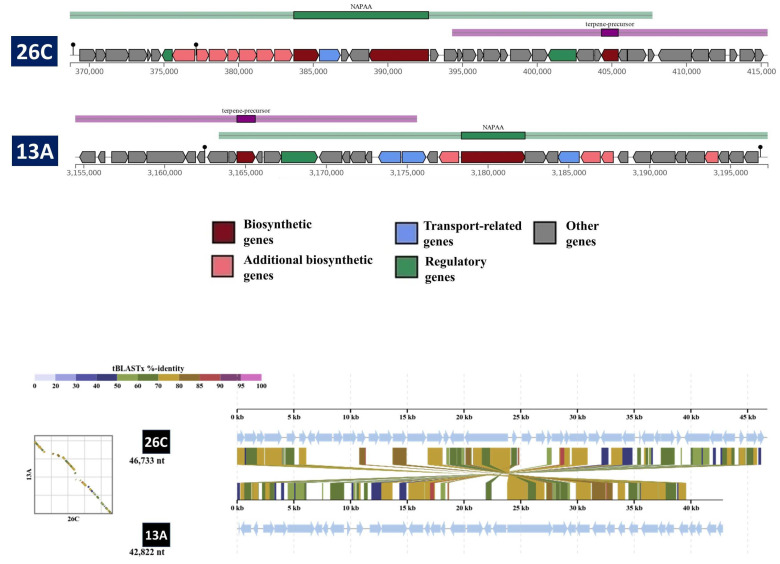
A comparison between the compositions of the NPAA cluster in *Brevibacterium luteolum* 26C and *Brevibacterium casei* 13A. The tblastx alignment between the two clusters was carried out by the DiGAlign server.

The antiSMASH identified a total of four and five BGCs within the genomes of *B. luteolum* 26C and *B. casei* 13A, respectively (Table 8). There was only one cluster highly similar to ε-poly-L-lysine, having high similarity between the two strains (Figure 7).

### 3.6. Comparative Orthologous Cluster Analysis

**Figure 8:**
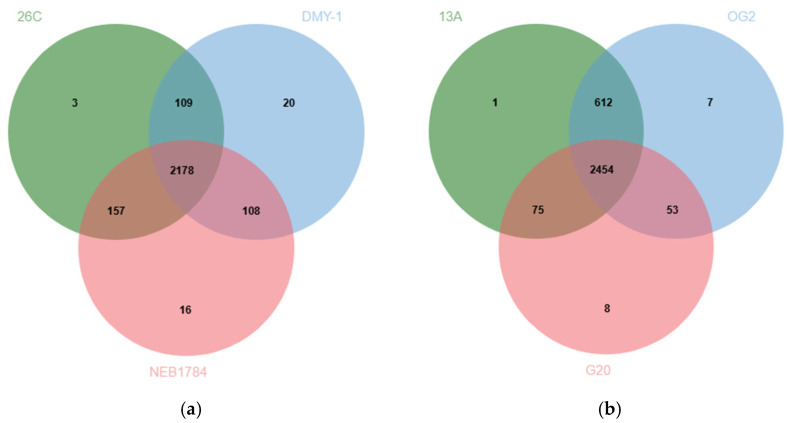
(**a**) A Venn diagram indicating the number of shared orthologous protein clusters between the genomes of *B. luteolum* strain 26C and the related *B. luteolum* strains DMY-1 and NEB1784. (**b**) A Venn diagram indicating the number of shared orthologous protein clusters between the genomes of *B. casei* strain 13A and the related *B. casei* strains OG2 and G20.

The orthologous clustering analysis conducted with OrthoVenn3 compared *B. luteolum* strain 26C against the related strains DMY-1 and NEB1784. A total of 2447 clusters were detected in 26C, 2415 in DMY-1, and 2459 in NEB1784. Among these, 2178 clusters were conserved across all three strains, representing the stable backbone of essential cellular functions. Strain 26C also harbored a distinct set of orthologous clusters associated with transposition and DNA restriction–modification systems (Figure 8a), which may contribute to genomic plasticity and defense against foreign genetic elements. In addition, the analysis highlighted 202 singleton genes specific to 26C, underscoring its distinct genomic potential. Of these, 61 were annotated as functional genes by Prokka, while the remaining genes were predicted as hypothetical proteins (Supplementary Table S1). The annotated genes spanned diverse categories, including metabolic enzymes (e.g., *ilvG*, *aceB*, *cadA*), regulators (e.g., *yurK*, *nanR*), transport systems for amino acids, peptides, and ions, as well as resistance determinants such as *abaF*, *hipA*, and *merA*, and multidrug efflux components (*mdtA*). A substantial proportion of these singletons corresponded to insertion sequences and transposases, reinforcing the observation of transposition-related clusters and highlighting the role of genome mobility in shaping the evolutionary trajectory of strain 26C. Together, these features suggest that strain 26C has evolved specialized capabilities for adaptability, resistance, and stress tolerance in its marine habitat.

Similarly, orthologous clustering analysis compared *B. casei* strain 13A with the related strains OG2 and G20. A total of 3142 clusters were detected in 13A, 3126 in OG2, and 2590 in G20. Among these, 2454 clusters were shared across all three strains, reflecting the conserved backbone of essential cellular functions. Strain 13A also contained a unique orthologous cluster associated with a membrane transporter protein *YrkJ* (Figure 8b). Moreover, OrthoVenn identified 277 singleton genes specific to 13A. Of these, 38 were annotated as functional proteins by Prokka, while the remaining genes were predicted as hypothetical proteins (Supplementary Table S2). The annotated proteins included metabolic enzymes (e.g., *poxB*, *sir*), regulators (e.g., *betI*, *slyA*, *cdhR*), and transport systems for amino acids, dicarboxylates, and ions (*yjeH*, *genK*, *sdcS*, *srpC*). Singletons corresponding to insertion sequences and transposases (IS3 family), alongside resistance determinants such as *arsC2* (arsenate resistance) and *bspRIM* (modification methylase), were identified.

## 4. Discussion

The Red Sea represents one of the most unique marine ecosystems globally, characterized by high salinity and pronounced thermal and nutrient gradients, which exert strong selective pressures on resident organisms [33]. These challenging environmental conditions support a remarkable diversity of marine microorganisms with specialized stress-tolerance and metabolic capabilities uniquely adapted to such stressors [34]. Within these microbial communities, the genus *Brevibacterium*, a member of the phylum Actinomycetota, has garnered increasing attention due to its biotechnological potential [35]. In this study, we present a comprehensive genomic analysis of two *Brevibacterium* strains, *B. luteolum* 26C and *B. casei* 13A, isolated from Red Sea sponges. Through the integration of phenotypic characterization, whole-genome sequencing, and genome mining, the study provides valuable insights into the ecological adaptation and biosynthetic potential of these marine-derived isolates.

Previous studies have reported the successful isolation of *Brevibacterium* sp. from various Red Sea environments, including sediments, coral reefs, and sponges [36,37,38]. However, to our knowledge, no prior study has confirmed the presence of *B. casei* or *B. luteolum* at the species level in the Red Sea. This study is the first to report and validate the occurrence of these two species in the Red Sea, based on whole-genome sequencing analysis. The ANI, AAI (96.77% and 96.31%, respectively, for *B. luteolum* 26C; 98.13% and 97.79%, respectively, for *B. casei* 13A), and dDDH (>70%) values meet accepted thresholds for species-level classification.

As an adaptation to the challenging marine environment, marine bacteria have been reported to possess biosynthetic and antistress genetic mechanisms that may not be present in their terrestrial counterparts [8,39]. These adaptations are generally acquired through horizontal gene transfer mediated by MGEs and GIs. Genomic islands are substantial, distinct segments of DNA within a genome, frequently acquired via HGT and commonly harboring genes that confer adaptive advantages to the host bacterium. In contrast, mobile genetic elements are smaller DNA sequences capable of relocating within or between genomes, such as plasmids and transposons [40,41]. Within this context, multiple GIs were identified in both strains (12 in 26C and 14 in 13A), many harboring genes associated with heavy metal resistance (e.g., cobalt, nickel, zinc, cadmium, and arsenic transporters). These genes likely contribute to the strains’ ability to withstand the fluctuating chemical and metal stressor characteristics of marine environments [42,43,44,45,46,47]. In addition, both strains also encoded multiple DNA repair mechanisms (e.g., helicases, glycosylases), suggesting mechanisms to counter genomic damage caused by marine environmental stressors such as UV radiation and desiccation, enhancing genome stability and ensuring survival under harsh marine conditions [48,49]. Notably, *B. casei* 13A harbored additional oxidative stress response genes (e.g., *ahpD*, thioredoxins), further supporting resilience under oxidative stress [50].

Beyond stress adaptation, the GIs in both strains encode genes associated with broad metabolic versatility such as those involved in allantoin catabolism (*allE*, *allB*), potentially facilitating nutrient acquisition and processing from the surrounding environment. Allantoin, derived from host excretion or from the surrounding seawater, represents a valuable nitrogen source in an oligotrophic environment. The ability to catabolize this compound suggests potential mutualistic benefits to the sponge host through nutrient recycling and broadens the nitrogen acquisition capability of these strains, especially in nitrogen-limited oligotrophic waters [51,52].

Genomic islands also encoded traits that may promote competitive adaptability and symbiotic roles within the sponge microbiome. Notably, quorum-quenching genes such as *ahlD* were identified, encoding an N-acyl-homoserine lactonase implicated in the disruption of microbial communication and biofilm formation [43,53]. Of particular ecological significance, GIs harboring multidrug transporter-related genes (e.g., *mdtL*) were identified in *B. casei* 13A, which may confer resilience against competitors’ antimicrobials, environmental toxins, and self-produced metabolites [54]. Furthermore, the genome of *B. casei* 13A harbored the complete *hcnABC* operon, responsible for hydrogen cyanide (HCN) biosynthesis, whereas *B. luteolum* 26C contained only the *hcnC* gene. Given the well-documented antimicrobial and antifungal activities of HCN, this difference may suggest that *B. casei* 13A plays a more pronounced role in microbial competition and host defense within the sponge microbiome [44,55,56,57]. These features collectively highlight the potential role of these strains, particularly *B. casei* 13A, as defensive symbionts in the highly competitive sponge microenvironment.

Moreover, the frequent occurrence of MGEs in both strains, particularly in *B. luteolum* 26C, which exhibited a higher abundance of integration, excision, and phage-associated genes compared to *B. casei* 13A, highlights the crucial role of horizontal gene transfer as a mechanism for rapid genomic evolution and the acquisition of new adaptive traits [58]. These features likely contribute to the ability of these strains to successfully colonize and persist within the dynamic and selective environment of marine sponge hosts. The greater representation of MGE-related genes in *B. luteolum* 26C further suggests a more dynamic or recently active history of genomic rearrangements and horizontal gene acquisition relative to *B. casei* 13A.

Both *B. luteolum* 26C and *B. casei* 13A strains exhibited considerable secondary metabolite biosynthetic potential. The antiSMASH analysis revealed four BGCs in *B. luteolum* 26C and five in *B. casei* 13A. Notably, both strains shared a cluster with high similarity to ε-poly-L-lysine biosynthesis, a compound with a broad-spectrum antimicrobial activity and food-preservative properties [59,60]. The presence of an ectoine biosynthesis module in *B. casei* 13A suggests a specialized role in osmoregulation, supporting its adaptation to marine ecosystems due to its role as a compatible solute and extremolyte [61].

The antimicrobial activity exhibited by *B. luteolum* 26C and *B. casei* 13A against *S. aureus*, *E. coli*, *and C. albicans* is strongly supported by their genomic profiles, as revealed by antiSMASH analysis, and the presence of GI-encoded secondary metabolite genes. These observations underscore the ecological role of *B. luteolum* 26C and *B. casei* 13A strains as active competitors and potential defensive associates within the sponge microbiome.

The comparative analysis with terrestrial counterparts showed potential genetic adaptation of strains 26C and 13A for the marine environment. For instance, the genome of *B. luteolum* 26C is characterized by several unique genetic elements, such as *merA*, *abaF*, and *mdtA*. The *merA* encodes the enzyme mercuric reductase, which is a key component of the *mer* operon, a gene system that provides resistance to mercury toxicity. The *MerA* enzyme converts highly toxic ionic mercury into a less toxic form. This process is of interest for bioremediation, as it allows for the cleanup of mercury-contaminated sites [62]. Furthermore, the ability of *Brevibacterium* to survive in these harsh marine conditions likely depends on its robust detoxification systems, including efflux pumps. The presence of several efflux-system-related genes, such as *abaF* and *mdtA*, suggests a strong adaptive potential in this marine strain compared to its terrestrial counterparts. This repertoire of efflux system genes enhances the strain’s ability to export harmful substances, like antibiotics and heavy metals, out of the bacterial cell, a mechanism that is known to confer multidrug resistance [63,64].

On the other hand, the orthologous clustering analysis of *B. casei* 13A in comparison with the terrestrial counterparts OG2 and G20 highlights both conserved genomic features and distinct adaptive traits, indicating that strain 13A may have developed specialized genomic traits supporting adaptability and stress tolerance in its marine environment. For instance, the presence of singletons related to metabolic enzymes (e.g., *poxB*, *sir*) and regulatory proteins (*betI*, *slyA*, *cdhR*) suggests potential modulation of central metabolism and transcriptional networks that could enhance metabolic flexibility under fluctuating marine conditions. In addition, transport-related genes (*yjeH*, *genK*, *sdcS*, *srpC*) point toward adaptations for nutrient acquisition in nutrient-variable seawater. Moreover, resistance determinants such as *arsC2* (arsenate resistance) and *bspRIM* (modification methylase) highlight the potential for resilience against toxic compounds and phage infection, traits that could provide selective advantages in complex marine microbial communities. Together, these features suggest that *B. casei* strain 13A has evolved specialized genomic strategies that balance the conservation of essential cellular machinery with the acquisition of novel functions, enabling successful adaptation to the marine environment [65,66,67].

## 5. Conclusions

This study provides a comprehensive genomic analysis of two *Brevibacterium* strains, *B. luteolum* 26C and *B. casei* 13A, isolated from Red Sea sponges. Whole-genome sequencing and functional annotation revealed their secondary metabolite gene clusters and genomic traits associated with adaptation to the region’s challenging physicochemical conditions. These include genes for osmotic regulation, hypersalinity tolerance, survival under nutrient limitation, and resistance to toxic compounds. Importantly, both strains exhibited antimicrobial activity against different pathogenic microorganisms, further emphasizing their ecological role. Collectively, these findings underscore the adaptive capacity of *Brevibacterium* in challenging marine environments and its potential biotechnological value.

## Figures and Tables

**Table 1:** Genomes used in the orthologous cluster analysis with corresponding NCBI accession numbers, isolation sources, genome sizes, and assembly levels.

Strain	Accession	Isolation Source	Genome Size	Assembly Level
*Brevibacterium luteolum* strain NEB1784	GCF_011462075.1	Contamination in NEB collection	3.2 Mb	Complete
*Brevibacterium luteolum* strain DMY-1	GCF_048832885.1	Livestock wastewater	3.1 Mb	Complete
*Brevibacterium casei* strain G20	GCA_019720815.1	Insect-associated	3.9 Mb	Complete
*Brevibacterium casei* strain OG2	GCF_002276605.1	Fermented milk	3.9 Mb	Contigs

**Table 2:** *B. luteolum* 26C and *B. casei* 13A colony morphology.

Feature	*B. luteolum* 26C	*B. casei* 13A
Colony Color	Yellowish	Grayish-white
Odor	Cheese-like	Cheese-like
Colony Texture and Shape	Smooth, rounded	Smooth, rounded
Microscopic Arrangement	Club-shaped bacilli in V/Y-shaped clumps	Diphtheroid-like rods
Incubation Time (on Marine Agar)	~1–2 days	~24 h (1 day)

**Table 3:** The results of the antimicrobial activity screening (measured as inhibition zone diameter in mm) of the metabolic extracts of strains 26C and 13A.

	*Staphylococcus aureus*	*Escherichia coli*	*Pseudomonas aeruginosa*	*Candida albicans*
26C	15	13	-	17
13A	17	15	-	15
Nystatin	-	-	-	30
Ampicillin	30	-	-	-
Imipenim	-	29	27	-
Cetazidim	-	28	20	-

**Table 4:** A summary of the genome annotation results of *Brevibacterium luteolum* 26C and *Brevibacterium casei* 13A.

	*B. luteolum* 26C		*B. casei* 13A	
	RAST	Prokka	RAST	Prokka
Total CDs	2672	2622	3449	3287
CDs with functional assignment	1786	1547	2416	1045
Hypothetical CDs	886	1075	1033	1371
rRNA	0	0	3	2
tRNA	45	52	49	53
EC assignment	711	994	849	1165

**Table 5:** A summary of the genomic islands (GIs) identified within the genome of *Brevibacterium luteolum* 26C and the genes identified within each GI, excluding hypothetical proteins.

Genomic Island Number	Size (bp)	Gene Name	Product
1	13,875	*allE*	(S)-ureidoglycine aminohydrolase
		*allB*	Allantoinase
		*gntR*	Putative D-xylose utilization operon transcriptional repressor
		*nikB*	Nickel transport system permease protein NikB
			Putative ABC transporter ATP-binding protein
			Putative ABC transporter ATP-binding protein
		*sgrR*	HTH-type transcriptional regulator SgrR
			8-oxoguanine deaminase
		*hyuE*	Hydantoin racemase
		*atzC*	N-isopropylammelide isopropyl amidohydrolase
2	16,179	*moeZ*	Putative adenylyltransferase/sulfurtransferase MoeZ
		*thiG*	Thiazole synthase
		*hcnC*	Hydrogen cyanide synthase subunit HcnC
		*mshD*	Mycothiol acetyltransferase
		*IS256*	IS256 family transposase ISBli22
		*menH*	2-succinyl-6-hydroxy-2, 4-cyclohexadiene-1-carboxylate synthase
		*mrpD*	Na(+)/H(+) antiporter subunit D
		*mrpC*	Na(+)/H(+) antiporter subunit C
		*ndhB*	NAD(P)H-quinone oxidoreductase subunit 2, chloroplastic
		*mshA*	D-inositol-3-phosphate glycosyltransferase
		*gpmB*	Phosphoglycerate mutase GpmB
		*rsfS*	Ribosomal silencing factor RsfS
3	11,816	*appA*	Oligopeptide-binding protein AppA
		*IS1380*	IS1380 family transposase IS1677
			Insertion element IS6110 uncharacterized 12.0 kDa protein
		*IS3*	IS3 family transposase ISBli28
		*IS256*	IS256 family transposase ISBli22
		*smpB*	SsrA-binding protein
4	10,747		Homocitrate synthase
		*IS3*	IS3 family transposase ISBli33
		*IS3*	IS3 family transposase ISBli33
			Insertion element IS6110 uncharacterized 12.0 kDa protein
		*IS5*	IS5 family transposase ISCgl5
		*gabT*	4-aminobutyrate aminotransferase
5	6619		Homocitrate synthase
		*IS3*	IS3 family transposase ISBli33
		*IS3*	IS3 family transposase ISBli33
			Insertion element IS6110 uncharacterized 12.0 kDa protein
6	6953	*cwhA*	N-acetylmuramoyl-L-alanine amidase A
		*merR1*	Mercuric resistance operon regulatory protein
		*merA*	Mercuric reductase
7	14,646	*insK*	Putative transposase InsK for insertion sequence element IS150
		*IS3*	IS3 family transposase ISBli17
		*IS3*	IS3 family transposase ISBli35
		*IS3*	IS3 family transposase ISAar26
		*IS3*	IS3 family transposase ISBli17
		*IS3*	IS3 family transposase ISBli35
		*cypB*	Peptidyl-prolyl cis-trans isomerase B
		*glpG*	Rhomboid protease GlpG
		*crgA*	Cell division protein CrgA
8	43,035	*scmP*	N-acetylcysteine deacetylase
		*ipuC*	Glutamate–isopropylamine ligase
		*murR*	HTH-type transcriptional regulator MurR
		*cadA*	Inducible lysine decarboxylase
			2-aminohexano-6-lactam racemase
		*pup*	Putrescine importer PuuP
		*uvrB*	UvrABC system protein B
		*nudG*	CTP pyrophosphohydrolase
		*fdhA*	Formate dehydrogenase subunit alpha
		*fdnG*	Formate dehydrogenase 2 subunit alpha (cytochrome c-553)
		*rsxB*	Ion-translocating oxidoreductase complex subunit B
		*recQ*	ATP-dependent DNA helicase RecQ
		*selD*	Selenide, water dikinase
		*selA*	L-seryl-tRNA(Sec) selenium transferase
		*selB*	Selenocysteine-specific elongation factor
		*Mct*	2-methylfumaryl-CoA isomerase
		*Meh*	Mesaconyl-C(4)-CoA hydratase
		*mmgC*	Acyl-CoA dehydrogenase
		*mmgC*	Acyl-CoA dehydrogenase
		*fumB*	Fumarate hydratase class I, anaerobic
		*yfdE*	Acetyl-CoA:oxalate CoA-transferase
		*glaR*	HTH-type transcriptional repressor GlaR
		*dctP*	C4-dicarboxylate-binding periplasmic protein DctP
		*dctM*	C4-dicarboxylate TRAP transporter large permease protein DctM
		*ISL3*	ISL3 family transposase ISPfr18
			S-(hydroxymethyl)mycothiol dehydrogenase
		*camD*	5-exo-hydroxycamphor dehydrogenase
		*ahlD*	N-acyl homoserine lactonase
9	6942	*fumB*	Fumarate hydratase class I, anaerobic
		*yfdE*	Acetyl-CoA:oxalate CoA-transferase
		*glaR*	HTH-type transcriptional repressor GlaR
		*dctP*	C4-dicarboxylate-binding periplasmic protein DctP
		*dctM*	C4-dicarboxylate TRAP transporter large permease protein DctM
10	9351		Insertion element IS6110 uncharacterized 12.0 kDa protein
		*IS3*	IS3 family transposase IS3501
			Insertion element IS6110 uncharacterized 12.0 kDa protein
11	27,035	*czcD*	Cadmium, cobalt and zinc/H(+)-K(+) antiporter
		*cmtR*	HTH-type transcriptional regulator CmtR
		*COQ5*	2-methoxy-6-polyprenyl-1,4-benzoquinol methylase, mitochondrial
		*lspA*	Lipoprotein signal peptidase
		*IS3*	IS3 family transposase ISBli33
			Insertion element IS6110 uncharacterized 12.0 kDa protein
		*IS3*	IS3 family transposase ISBli33
		*resA*	Thiol-disulfide oxidoreductase ResA
		*czcD*	Cadmium, cobalt, and zinc/H(+)-K(+) antiporter
		*cseB*	Transcriptional regulatory protein CseB
		*sasA*	Adaptive-response sensory-kinase SasA
		*copB*	Copper-exporting P-type ATPase B
		*Idi*	Isopentenyl-diphosphate Delta-isomerase
12	6694	*cmtR*	HTH-type transcriptional regulator CmtR
		*COQ5*	2-methoxy-6-polyprenyl-1,4-benzoquinol methylase, mitochondrial
		*lspA*	Lipoprotein signal peptidase
		*IS3*	IS3 family transposase ISBli33
			Insertion element IS6110 uncharacterized 12.0 kDa protein
		*IS3*	IS3 family transposase ISBli33

**Table 6:** A summary of the genomic islands (GIs) identified within the genome of *Brevibacterium casei* 13A and the genes identified within each GI, excluding hypothetical proteins.

Genomic Island Number	Size (bp)	Gene Name	Product
1	4136	*yknY_1*	Putative ABC transporter ATP-binding protein YknY
2	12,733	*bspRIM*	Modification methylase BspRI
3	11,620	*yegS_2*	Lipid kinase YegS
			Insertion element IS6110 uncharacterized 12.0 kDa protein
		*IS3*	IS3 family transposase ISBli25
		*yegS_1*	lipid kinase YegS
		*serS*	Serine–tRNA ligase
4	27,730	*prfB*	Peptide chain release factor 2
		*ftsE*	Cell division ATP-binding protein FtsE
		*ftsX*	Cell division protein FtsX
		*smpB*	SsrA-binding protein
			Putative prophage phiRv2 integrase
		*arsC2*	Arsenate-mycothiol transferase ArsC2
		*srpC_3*	Putative chromate transport protein
		*xerC_2*	Tyrosine recombinase XerC
		*rhaR_3*	HTH-type transcriptional activator RhaR
		*copZ_2*	Copper chaperone CopZ
		*ctpA*	Copper-exporting P-type ATPase
5	7010	*rhaR_3*	HTH-type transcriptional activator RhaR
6	4036	*fpg1_2*	Formamidopyrimidine-DNA glycosylase 1
		*merR1*	Mercuric resistance operon regulatory protein
		*merA*	Mercuric reductase
		*merB*	Alkylmercury lyase
7	6926	*lspA_2*	Lipoprotein signal peptidase
		*ctpG*	Putative cation-transporting ATPase G
		*cmtR_1*	HTH-type transcriptional regulator CmtR
		*cueR*	HTH-type transcriptional regulator CueR
		*fpg1_1*	Formamidopyrimidine-DNA glycosylase 1
		*srpC_1*	Putative chromate transport protein
8	17,165	*rpoD_2*	RNA polymerase sigma factor RpoD
		*addA*	ATP-dependent helicase/nuclease subunit A
9	31,029	*IS3*	IS3 family transposase ISBli35
		*IS3*	IS3 family transposase ISBli35
		*recD2*	ATP-dependent RecD-like DNA helicase
		*uvrB_2*	UvrABC system protein B
		*yfeO*	Putative ion-transport protein YfeO
11	10,728		Putative prophage phiRv2 integrase
		*metF*	5,10-methylenetetrahydrofolate reductase
12	56,608	*yidC_2*	Membrane protein insertase YidC
		*cpdA_2*	3′,5′-cyclic adenosine monophosphate phosphodiesterase CpdA
			Sulfurtransferase
		*gloB_3*	Hydroxyacylglutathione hydrolase
		*gloB_4*	Hydroxyacylglutathione hydrolase
		*ricR_2*	Copper-sensing transcriptional repressor RicR
		*hcaD*	3-phenylpropionate/cinnamic acid dioxygenase ferredoxin--NAD(+) reductase component
		*mdtL*	Multidrug resistance protein MdtL
			Ferredoxin
		*ydhK*	Putative protein YdhK
		*copB_2*	Copper-exporting P-type ATPase B
		*ricR_3*	Copper-sensing transcriptional repressor RicR
		*ahpD_2*	Alkyl hydroperoxide reductase AhpD
		*czcD_2*	Cadmium, cobalt, and zinc/H(+)-K(+) antiporter
		*trxA_3*	Thioredoxin 1
		*IS3*	IS3 family transposase ISBli33
		*IS3*	IS3 family transposase ISBli33
		*ISL3*	ISL3 family transposase ISAar42
		*ISL3*	ISL3 family transposase ISBli30
		*trxC_2*	Putative thioredoxin 2
		*gloB_5*	Hydroxyacylglutathione hydrolase
		*ygaP*	Inner membrane protein YgaP
		*tnpR*	Transposon Tn3 resolvase
		*cmtR_2*	HTH-type transcriptional regulator CmtR
13	17,769	*ISL3*	ISL3 family transposase ISAar42
		*ISL3*	ISL3 family transposase ISBli30
		*trxC_2*	Putative thioredoxin 2
14	6653	*tnpR*	Transposon Tn3 resolvase
		*cmtR_2*	HTH-type transcriptional regulator CmtR

**Table 7:** A summary of the mobile-genetic-element-related genes identified within the genomes of *Brevibacterium luteolum* 26C and *Brevibacterium casei* 13A.

Category	Number of Genes	
	*B. luteolum* 26C	*B. casei* 13A
Integration/excision	28	7
Replication/recombination/repair	17	23
Phage	12	9
Stability/transfer/defense	7	4
Transfer	3	6
Total	67	49

**Table 8:** Biosynthetic gene clusters (BGCs) identified within the genomes of *Brevibacterium luteolum* 26C and *Brevibacterium casei* 13A using antiSMASH version 8.

Cluster	Type	Size (bp)	Most Similar Known Cluster	Similarity	26C	13A
**1**	NAGGN	15,198	-	-	✔	✘
**2**	NAPAA	39,023–42,821	ε-poly-L-lysine	High	✔	✔
**3**	Tropodithietic acid	42,257	5-dimethylallylindole-3-acetonitrile	Medium	✔	✘
**4**	Terpene	25,204	Carotenoid	Medium	✘	✔
**5**	Terpene	23,296	Carotenoid	Low	✔	✘
**6**	Hydrogen cyanide	13,141	-	-	✘	✔
**7**	Ectoine	10,401	Ectoine	Medium	✘	✔
**8**	NI-siderophore	30,594	FW0622	Low	✘	✔

Note: The symbol (✔)means present, while the symbol (✘) means absent.

## Data Availability

The original data presented in the study are openly available in the National Center for Biotechnology Information (NCBI) GenBank repository. For *Brevibacterium casei* strain 13A, the sequence data are accessible via BioProject accession PRJNA1280801, Biosample accession SAMN49535178, SRA accession SRR34103316, and Genome accession GCA_051216575.1. For *Brevibacterium luteolum* strain 26C, the sequence data are accessible via BioProject accession PRJNA1280809, Biosample accession SAMN49535213, SRA accession SRR34103435, and Genome accession GCA_051216595.1.

## Supplementary Materials

The following supporting information can be downloaded at: https://www.mdpi.com/article/10.3390/biology14091271/s1, Table S1: Functional annotation of *B. luteolum* strain 26C-specific singletons identified by orthologous cluster analysis using OrthoVenn3. Table S2: Functional annotation of *B. casei* strain 13A-specific singletons identified by orthologous cluster analysis using OrthoVenn3.

## Abbreviations

The following abbreviations are used in this manuscript:

AAI	Average Amino Acid Identity
ANI	Average Nucleotide Identity
ANIm	ANI using MUMmer alignment
BGC(s)	Biosynthetic Gene Cluster(s)
CDs/CDSs	Coding Sequences
dDDH	Digital DNA–DNA Hybridization
DMSO	Dimethyl Sulfoxide
GI(s)	Genomic Island(s)
GTDB	Genome Taxonomy Database
GTDB-Tk	GTDB Toolkit
MGE(s)	Mobile Genetic Element(s)
NSW	Natural Seawater
Prokka	Rapid Prokaryotic Genome Annotation
TYGS	Type Strain Genome Server
WGS	Whole-Genome Sequencing
