# Supplementary material for: Comprehensive Genome Analysis of Two Bioactive Brevibacterium Strains Isolated from Marine Sponges from the Red Sea
Source: Biology (Basel). 2025 Sep 15;14(9):1271. doi: 10.3390/biology14091271 (PMC12467314; doi:10.3390/biology14091271)
Supplement: Supplementary file 1 [file biology-14-01271-s001.zip › biology-3852267-supplementary.pdf]

Supplementary Materials

Comprehensive Genome Analysis of Two Bioactive Brevibacterium Isolates Associated with Marine Sponges from the Red Sea

Yehia S. Mohamed [1,2]*, Samar M. Solyman [3,4]*, Abdelrahman M. Sedeek [5], Hasnaa Lotfy Kamel [3] and Manar El Samak [4]

[1] Department of Pathological Sciences, College of Medicine, Ajman University, Ajman, UAE
[2] Department of Microbiology and Immunology, Faculty of Pharmacy (boys), Al-Azhar University, Cairo, Egypt.
[3] Department of Microbiology & Immunology, Faculty of Pharmacy, Sinai University- Elkantara branches, Ismailia, Egypt.
[4] Department of Microbiology & Immunology, Faculty of Pharmacy, Suez Canal University, Ismailia, Egypt.
[5] Department of Microbiology & Immunology, Faculty of Pharmacy, Galala University, New Galala City, Suez 43511, Egypt
* Correspondence: Dr. Yehia S. Mohamed (y.mohamed@ajman.ac.ae), and Prof. Samar M. Solyman (Samar.mansour@su.edu.eg)

Table S1. Functional annotation of *B. luteolum* strain 26C-specific singletons identified by orthologous cluster analysis using OrthoVenn3**.**

Gene	Product
rlmCD	23S rRNA (uracil-C(5))-methyltransferase RlmCD
thiC	Phosphomethylpyrimidine synthase
hyuC	N-carbamoyl-L-amino-acid hydrolase
gtfA	UDP-N-acetylglucosamine--peptide N-acetylglucosaminyltransferase GtfA
namA	NADPH dehydrogenase
nfdA	N-substituted formamide deformylase
ilvG	Acetolactate synthase isozyme 2 large subunit
aceB	Malate synthase A
allB	Allantoinase
recD2	ATP-dependent RecD-like DNA helicase
pdxA2	D-erythronate 4-phosphate dehydrogenase
denK	D-erythronate kinase
scmP	N-acetylcysteine deacetylase
ipuC	Glutamate--isopropylamine ligase
cadA	Inducible lysine decarboxylase
	2-aminohexano-6-lactam racemase
fdhA	Formate dehydrogenase subunit alpha
fdnG	Formate dehydrogenase, nitrate-inducible, major subunit
mct	2-methylfumaryl-CoA isomerase
meh	Mesaconyl-C(4)-CoA hydratase
fumB	Fumarate hydratase class I, anaerobic
	S-(hydroxymethyl)mycothiol dehydrogenase
camD	5-exo-hydroxycamphor dehydrogenase
ahlD	N-acyl homoserine lactonase
tgnC	(Z)-2-((N-methylformamido)methylene)-5-hydroxybutyrolactone dehydrogenase
dag	N-acyl-D-glutamate deacylase
rutD	Putative aminoacrylate hydrolase RutD
yurK	Putative HTH-type transcriptional regulator YurK
rapA	RNA polymerase-associated protein RapA
betI	HTH-type transcriptional regulator BetI
srlR	Glucitol operon repressor
nanR	HTH-type transcriptional repressor NanR
kdpE	Transcriptional regulatory protein KdpE
rcsC	Sensor histidine kinase RcsC
hipA	Serine/threonine-protein kinase toxin HipA
abaF	Fosfomycin resistance protein AbaF
merA	Mercuric reductase
mdtA	Multidrug resistance protein MdtA

putP	High-affinity proline transporter PutP
gntU	Low-affinity gluconate transporter
puuP	Putrescine importer PuuP
dctP	C4-dicarboxylate-binding periplasmic protein DctP
kdpC	Potassium-transporting ATPase KdpC subunit
kdpB	Potassium-transporting ATPase ATP-binding subunit
kdpA	Potassium-transporting ATPase potassium-binding subunit
rhtA	Threonine/homoserine exporter RhtA
gsiC	Glutathione transport system permease protein GsiC
gsiD	Glutathione transport system permease protein GsiD
dppD	Dipeptide transport ATP-binding protein DppD
oppF	Oligopeptide transport ATP-binding protein OppF
appA	Oligopeptide-binding protein AppA
	IS1380 family transposase IS1677
	IS6110 insertion element protein
	IS256 family transposase ISBli22
	IS5 family transposase ISCgl5
insK	Putative transposase InsK
	IS3 family transposase ISBli17
	IS3 family transposase ISBli35
	IS6110 insertion element protein
	Putative protein (COG2114)
	Putative protein (COG3177)

Table S2. Functional annotation of *B. casei* strain 13A-specific singletons identified by orthologous cluster analysis using OrthoVenn3.

Gene	Product
poxB	Pyruvate dehydrogenase [ubiquinone]
tgnC	(Z)-2-((N-methylformamido)methylene)-5-hydroxybutyrolactone dehydrogenase
cypM	Cypemycin N-terminal methyltransferase
	putative methyltransferase
	putative methyltransferase
yeaX	Carnitine monooxygenase reductase subunit
fpg1	Formamidopyrimidine-DNA glycosylase 1
ftsH	ATP-dependent zinc metalloprotease FtsH
aroK	Shikimate kinase
oxyE	6-methylpretetramide 4-monooxygenase
sir	Sulfite reductase [ferredoxin]
gloB	Hydroxyacylglutathione hydrolase
	Putative prophage phiRv2 integrase
betI	HTH-type transcriptional regulator BetI
slyA	Transcriptional regulator SlyA
cdhR	HTH-type transcriptional regulator CdhR
rpoD	RNA polymerase sigma factor RpoD
rraA	Regulator of ribonuclease activity A
	putative HTH-type transcriptional regulator
arsC2	Arsenate-mycothiol transferase ArsC2
srpC	putative chromate transport protein
nepI	Purine ribonucleoside efflux pump NepI
	putative ABC transporter ATP-binding protein
yjeH	L-methionine/branched-chain amino acid exporter YjeH
dctM	C4-dicarboxylate TRAP transporter large permease protein DctM
genK	Gentisate transporter
sdcS	Sodium-dependent dicarboxylate transporter SdcS
	IS3 family transposase ISGmo1
	IS3 family transposase ISBli35
bspRIM	Modification methylase BspRI
hisC	Histidinol-phosphate aminotransferase
	Cytochrome p450 CYP199A2
addA	ATP-dependent helicase/nuclease subunit A
ftsP	Cell division protein FtsP
uvrB	UvrABC system protein B
trxC	Putative thioredoxin 2
ygaP	Inner membrane protein YgaP
	putative protein
